# Sub-acute combined degeneration with an initially normal level of vitamin B12: a case report

**DOI:** 10.4076/1757-1626-2-6944

**Published:** 2009-08-05

**Authors:** Fadi Makdsi, Tareck Kadrie

**Affiliations:** Department of Medicine, University of Tennessee, College of Medicine975 East Third Street, Suite 94, Chattanooga, TN 37403USA

## Abstract

**Introduction:**

The neurological manifestation of vitamin B12 deficiency can occur as a result of peripheral nerve pathology or lateral and posterior column involvement, also known as “sub-acute combined degeneration”. This case report demonstrates an unusual presentation of SCD with normal B12 level.

**Case presentation:**

A 39-year-old man was referred to the outpatient neurology clinic with a two month history of distal upper extremities numbness and fine motor movement difficulties. Initial vitamin B12 level was normal. A repeat MR imaging of the cervical and thoracic spine showed extensive posterior cervical cord flame-shaped lesions. His repeat vitamin B12 level was 41 pg/ml (normal; 200 ph/ml). He received monthly injections of vitamin B12. After six months his symptoms were resolved and his repeat spinal MRI showed resolution of the previous lesions.

**Conclusion:**

We recommend that every patient presenting with numbness and lesions on a spinal MR imaging should have their vitamin B12 level checked.

## Introduction

Vitamin B12 deficiency is a common disorder. Its manifestations can range from asymptomatic to megaloblastic anemia. A wide spectrum of neurological symptoms can also occur as a result of peripheral nerve pathology or lateral and posterior column involvement, also known as “sub-acute combined degeneration”. We present a patient with progressive neurological symptoms including numbness and fine motor movement difficulties. His borderline level of serum vitamin B12 initially led to an extensive workup and relative delay in the diagnosis of vitamin B12 deficiency as the cause of his symptoms. This case report demonstrates an unusual presentation of SCD with normal B12 level and emphasizes the importance of measuring methylmalonic acid and homocysteine if vitamin B12 level is in the low normal limit.

## Case presentation

**Figure 1.**
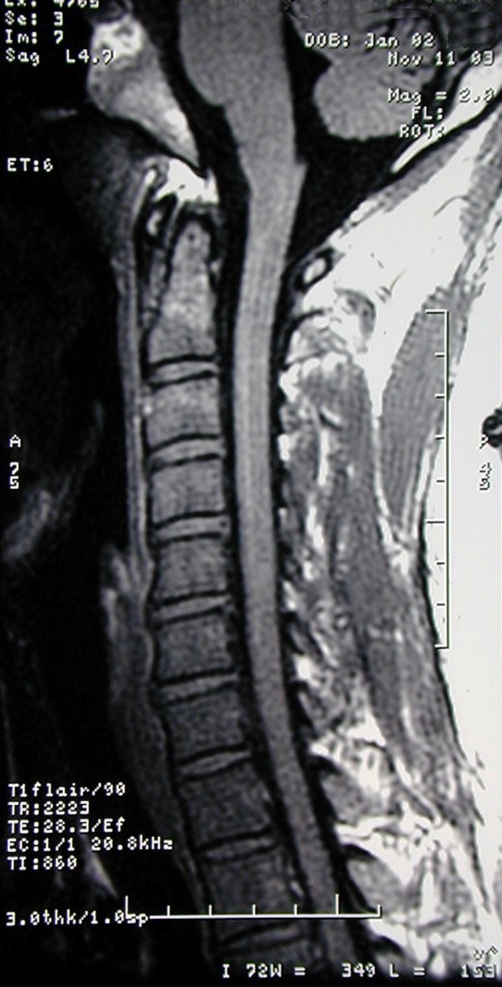
Normal cervical spine T1 weighted MRI.

**Figure 2.**
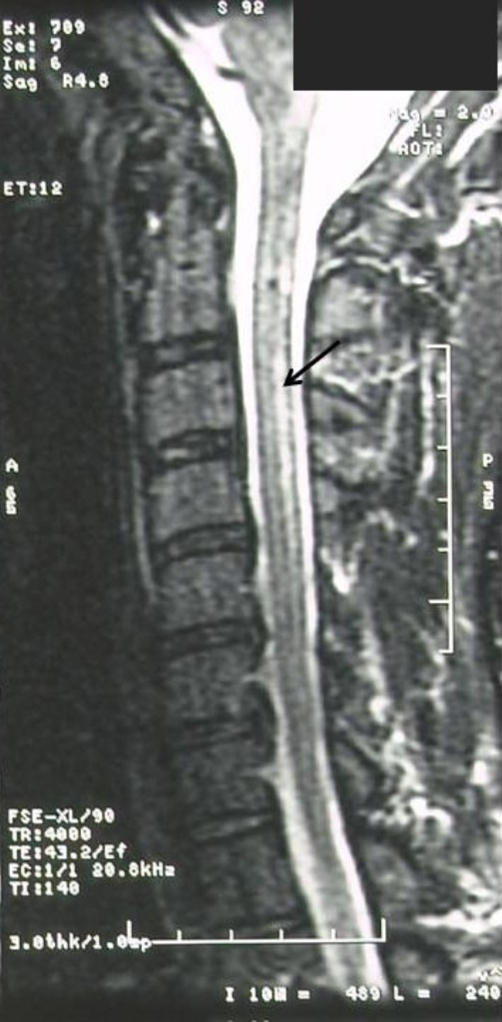
Flamed-shaped area of increased signal intensity within the posterior column of the cervical spine on the sagittal T2 weighted MRI.

**Figure 3.**
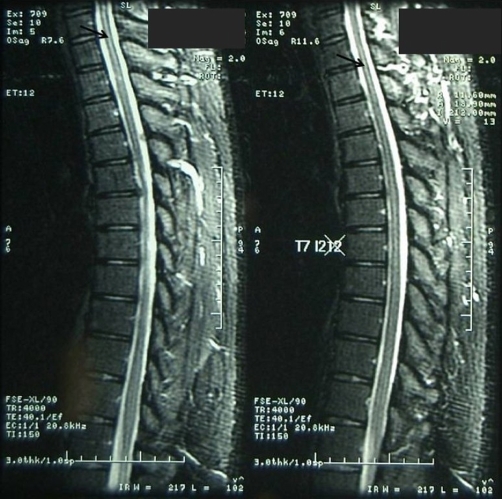
Flamed-shaped area of increased signal intensity within the posterior column of the cervical and thoracic spine on the sagittal T2 weighted MRI.

**Figure 4.**
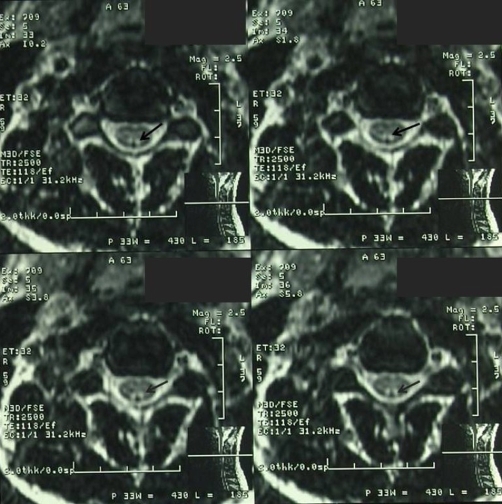
Increased signal intensity within the posterior column of the cervical spine on the axial T2 weighted MRI.

A 39-year-old African American man was referred to the outpatient neurology clinic with a two month history of distal upper extremities numbness and fine motor movement difficulties. He denied any neck pain, lower extremity symptoms, vision loss or any other neurological deficit. His physical exam was normal, including a detailed neurological exam. Laboratory evaluation six weeks prior had revealed normal basic chemistry profile, HGB 11.9 g/dl (MCV, and MCH values were not available at that time), and B12 203 pg/ml (normal; >180 pg/ml). He also underwent an EMG and nerve conduction study which revealed mild bilateral carpal tunnel syndrome. Other investigations conducted beforehand included MR imaging of the cervical spine three weeks prior, which revealed increased signal intensity within the core of the spinal cord at the level of C 3-4 on T2 weighted sagittal images. Axial images were not obtained at this level and contrast was not used in this study. A repeat of his cervical and thoracic MR imaging showed extensive posterior cervical cord flame-shaped lesions on T2 weighted images (Figure 1,Figure 2,Figure 3,Figure 4). Extensive laboratory evaluation was unremarkable including: basic chemistry profile, erythrocyte sedimentation rate, TSH, angiotensin converting enzyme, hemoglobin A1C, RPR, ANA, RF, serum protein electrophoresis, iron studies, Lyme IgG antibodies and heavy metals screen. His repeat Vitamin B12 level was 41 pg/ml, Methylmalonic acid 5.7 umol/l (normal; <0.4 umol/l), Homocysteine >50 nmol/l (normal; 5-18 nmol/l), folate 12.7 ng/ml. Pernicious anemia was suspected and investigation revealed positive intrinsic factor antibodies and negative anti-parietal antibodies. He received monthly injections of vitamin B12 replacement therapy. After six months his symptoms were resolved and his repeat spinal MRI showed resolution of the previous lesions.

## Discussion

The myelopathy in vitamin B12 deficiency, also known as sub-acute combined degeneration (SCD), is caused by demyelination of the posterior column and the lateral tracts of the cervical and thoracic spine. Similar to our patient, the most common initial complaint is paresthesias and physical examination may be normal.

The MRI finding of patients with sub-acute combined degeneration may reveal a regional T2 hyperintensity mainly in the thoracic and cervical posterior column. The differential diagnosis of an MRI with spinal posterior cord lesion is vast and can include multiple sclerosis, infectious myelitis, spinal infarction, and malignancies. Sub-acute combined degeneration demonstrates contiguous involvement in the posterior column of multiple segments of the cord. However, in other etiologies of demyelinating lesions, the spinal lesions are scattered and often do not involve more than two vertebral bodies in length [1]. Furthermore, as in our patient, the improvement of neurological symptoms and the resolution of the MR imaging abnormalities following his vitamin B12 replacement supports this diagnosis.

It is important to distinguish B12 deficiency from other causes of myelopathy as it is curable and early detection is necessary for full clinical recovery. Checking serum B12 is used to confirm the diagnosis. However, when the level of vitamin B12 is in the low normal range, high serum levels of methylmalonic acid and homocysteine should be used to coin the diagnosis. Spinal MR imaging with features of SCD can also help detect early disease. Once the diagnosis is confirmed, the treatment of a vitamin B12 injection should be started as early as possible to avoid irreversible neurological damage.

Our patient presented with unexplained numbness and fine motor movement difficulties. His evaluation was initially challenging because of the normal level of vitamin B12. Multiple investigations and delayed diagnosis might have been prevented if his metabolite was measured first. Three weeks later, his spinal MRI suggested the diagnosis of myelopathy and the diagnosis of vitamin B12 deficiency was confirmed by repeating the serum vitamin B12 level and checking the levels of methylmalonic acid and homocysteine.

## Conclusion

We recommend that vitamin B12 level should be checked in every patient presenting with numbness and particularly in those with unexplained lesions on a spinal MR imaging. Furthermore, methylmalonic acid and homocysteine should also be measured if vitamin B12 level is in the low normal limit. MR imaging may be a useful addition to the clinical assessment and in monitoring the efficacy of treatment of vitamin B12 deficiency.

## Abbreviations

EGD: esophagogastroduodenoscopy
EMG: electromyography
MRI: magnetic resonance imaging
SCD: sub-acute combined degeneration
